# Dual-Action Sutures: Chlorhexidine and Dexamethasone for Infection Control and Inflammation Suppression

**DOI:** 10.3390/molecules31071200

**Published:** 2026-04-04

**Authors:** Brisa Guadalupe Hernández-Ramírez, Giovanni Palomino-Vizcaino, Lilia Angélica Hurtado-Ayala, Jonathan Vincent Lopez-Baena, Hebrón Vazquez-Estudillo, Arturo Estolano-Cobián, Teresa G. Rodriguez-Tellez, Héctor Milla-Hinojosa, José Manuel Cornejo-Bravo, Kenia Palomino-Vizcaino, Héctor Magaña

**Affiliations:** 1 Faculty of Chemical Sciences and Engineering, Autonomous University of Baja California, University Boulevard 14418, International Industrial Park Tijuana, Tijuana 22390, Mexico; brisa.hernandez@uabc.edu.mx (B.G.H.-R.); lilyhurtado@uabc.edu.mx (L.A.H.-A.); vlopez16@uabc.edu.mx (J.V.L.-B.); hebron.vazquez@uabc.edu.mx (H.V.-E.); estolano.arturo@uabc.edu.mx (A.E.-C.); teresa.rodriguez.tellez@uabc.edu.mx (T.G.R.-T.); jmcornejo@uabc.edu.mx (J.M.C.-B.); kenia.palomino@uabc.edu.mx (K.P.-V.); 2 Faculty of Health Sciences, Autonomous University of Baja California, University Boulevard 1000, Valle de Las Palmas, Tijuana 22260, Mexico; gpalomino@uabc.edu.mx; 3 Tjplast Outpatient, 9288 Heroes Avenue, Rio Zone, Tijuana 22010, Mexico; drmilla@tjplast.com

**Keywords:** chlorhexidine, dexamethasone, surgical suture, surgical site infection, inhibition zone test, drug release system, functionalized suture, drug-eluting suture, dual drug-eluting suture

## Abstract

Surgical site infections (SSIs) remain a major clinical challenge, particularly due to bacterial adhesion and biofilm formation on suture materials. In this study, we developed a dual drug-eluting suture incorporating chlorhexidine (CHX) and dexamethasone (DEX), with lauric acid used as a binding agent to enhance drug adhesion. The exact composition of the system was CHX/DEX/Lauric Acid, designed to enable localized delivery of both therapeutic agents at the implantation site. Vicryl sutures were dip-coated and characterized by means of FTIR-ATR and HPLC to confirm drug incorporation and release. Mechanical integrity was preserved, with no significant difference in tensile strength between coated and uncoated sutures. Antimicrobial activity was confirmed against Gram-positive and -negative bacteria, including methicillin-resistant *Staphylococcus aureus* (MRSA), in addition to the yeast *Candida albicans*. Cell viability assays demonstrated acceptable biocompatibility, with values exceeding 70%. These findings support the potential of dual-functionalized sutures to reduce SSIs and modulate inflammation, offering a promising strategy for improving postoperative outcomes.

## 1. Introduction

A surgical site infection (SSI) is a common type of healthcare-associated infection (HAI) that occurs at the site of the surgical incision. This infection can occur within 30 days post-procedure or up to one year following the insertion of a medical implant [1,2]. In the U.S., the Centers for Disease Control and Prevention (CDC) reports that approximately 1 in 31 patients acquires an HAI. Furthermore, the annual cost of treating SSIs is estimated at 3.3 billion, making SSIs one of the more expensive HAIs [3]. The World Health Organization (WHO) has reported that up to 11% of patients in low- and middle-income countries develop an SSI [4].

After injury to the skin, the exposed subendothelium, collagen, and tissue factor activate platelet aggregation, leading to degradation and the release of chemotactic factors (chemokines) and growth factors (GFs) that contribute to clot formation. The aforementioned processes contribute to successful hemostasis [5]. The wound-healing process is a multifaceted process governed by sequential yet overlapping phases, including the hemostasis/inflammation phase, the proliferation phase, and the remodeling phase [6]. Infection occurs when virulence factors expressed by one or more microorganisms in a wound outcompete the host’s natural immune system, and subsequent invasion and dissemination of microorganisms in viable tissue provokes a series of local and systemic host responses [7]. Characteristic local responses include purulent discharge or painful spreading erythema indicative of cellulitis around a wound [8].

Sutures are medical devices used to hold tissue edges together to achieve wound closure, seal surgery sites, or compress blood vessels to achieve hemostasis [9]. Suture threads are classified by structure as monofilament, multifilament (braided), and barbed and by their biodegradability as non-absorbable or absorbable, which degrade through hydrolysis or proteolytically, typically in 60–90 days [9]. Monofilament sutures possess a uniform surface with low capillarity, in contrast to braided sutures, in which several strands are braided together, forming a multifilament suture with higher capillarity, making it less efficient in handling infections than monofilament sutures [10,11]. Braided sutures exhibit greater flexibility and enhanced tensile strength compared to non-braided types, which increases wound closure efficiency but has been associated with higher rates of SSI. This increase is linked to the presence of interstitial spaces between fibers, which facilitate bacterial attachment and create microenvironments that may protect microorganisms from phagocytic immune cells such as neutrophils and macrophages, contributing to a slower rate of bacterial clearance [12,13].

Surgical sutures are susceptible to contamination from environmental sources or the patient’s own microbiota during wound healing [14]. In one study, a suspension of 10^5^ UCF/mL *Staphylococcus aureus* bacteria failed to promote or develop local infection on a wound; in comparison, the presence of a suture potentiated bacterial growth and caused infection in mice [15]. Several preventive strategies, such as skin antisepsis, oxygen supplementation, intranasal decolonization, and antimicrobial prophylaxis, have been employed to reduce the risk of SSI. Despite these efforts, sutures remain key sites for bacterial adhesion and potential sources of wound infection [16]. The most common pathogens are members of the skin microbiota, such as *Staphylococcus aureus* and *Streptococcus* species, or intestinal microbiota, including *Enterococcus* species and *Escherichia coli* [16].

The results of in vivo suture-adherence assays using radiolabeled bacteria highlighted significant differences in the affinity of bacteria (*S. aureus*, *E. coli*, *S. marcescens*, *S. dysenteriae*, and *B. fragilis*) for various suture materials. A braided suture exhibited the greatest level of binding to the bacteria [15].

The microbiological profiles of SSI are related to the type and anatomical site of the procedure. *S. aureus* is commonly associated with infections following cardiac, neurosurgical, breast, and orthopedic procedures, in addition to being detected in patients receiving grafts, prostheses, or implants. In contrast, infections caused by Gram-negative bacilli are more often associated with appendectomy and colorectal, urologic, obstetric, and gynecologic surgeries [1]. Notably, *S. aureus* is the most common cause of infection, accounting for 24% of non-superficial infections. It can form biofilms on sutures, particularly on braided materials such as polyglactin 910, after 24 h [16,17]. This increased adherence is attributed not only to the braided structure but also to the material’s specific physicochemical properties; microorganisms are more likely to attach to hydrophobic sutures and remain stable under low-humidity conditions [18,19].

Chlorhexidine (CHX) is a cationic antiseptic that disrupts cells due to its biguanide structure. It exhibits a broad spectrum of activity against both Gram-positive and Gram-negative bacteria, with it being included in the NICE 2019 and WHO 2018 guidelines to reduce SSI rates, due to its limited development of bacterial resistance [20,21]. For this reason, CHX has been used to functionalize surgical sutures, thereby conferring them with antimicrobial properties. The combined device, integrating sutures and CHX, serves the dual function of closing a wound and delivering antibiotics to prevent or delay bacterial attachment and consequent biofilm formation [22].

Existing evidence demonstrates that the use of antimicrobial sutures can reduce the incidence of SSI and reduce dependence on antibiotics [23]. As an alternative to avoid polypharmacy, treating multiple targets simultaneously using one device is an emerging field of research [24].

Additionally, inflammation plays a key role in eliminating contaminating microorganisms and in the wound-healing process [25]. The contaminating bacterium or its components, such as endotoxins, induce the release of cytokines from inflammatory cells such as IL-1, IL-6, and TNF-α, which leads to overexpression of matrix metalloproteinases (MMP-1, MMP-2, MMP-8, and MMP-9), resulting in degradation of essential growth factors such as Transforming Growth Factor-β (TGF)-β and Platelet-Derived Growth Factor (PDGF) in healing chronic wounds, prolonging the inflammatory state that potentially leads to cell necrosis [26]. To optimize wound healing, it is essential to maintain a balance between the inflammatory and reparative phases [27,28]. While moderate inflammation contributes to the healing process, a prolonged and excessive inflammatory response can impair wound healing; in contrast, well-controlled inflammation often promotes wound healing [29]. The safety of anti-inflammatory agents, such as dexamethasone, has been evaluated for perioperative use [30].

Dexamethasone (DEX) has potent anti-inflammatory and immunosuppressive effects [31]. In healthy mice, a single dose of dexamethasone reduces pro-inflammatory cytokine levels, resulting in an insufficient number of macrophages during the healing process [32]. This incomplete inflammatory response results in an ongoing initial inflammatory phase rather than progression into the proliferative phase of wound healing, as observed in healthy mice [32]. In contrast, in septic mice, dexamethasone alleviates excessive inflammation, maintaining the balance between M1/M2 macrophages during the early and late phases of healing, promoting wound healing without significantly increasing SSI risk [32,33].

In humans, long-term treatment using dexamethasone is associated with side effects, such as reduced sleep quality, high risk of infection, and early postoperative hyperglycemia, leading to impaired wound healing and increased anastomotic drainage [34,35]. The prophylactic perioperative use of one dose of dexamethasone shows that it decreases the probability of postoperative infection; it remains a source of debate, however, as to whether it increases the risk of impaired wound healing [36]. In a study in which the authors evaluate the use and safety of single low-dose dexamethasone in the perioperative period of gynecological surgeries, it did not result in increased risk of SSI [37]. In a randomized single-blind study, patients with uninfected mandibular fractures who received 30 mg of dexamethasone perioperatively did not observe a significant difference in the incidence of impaired wound healing compared to the control [36]. The results of a trial including high-risk patients, such as diabetics, demonstrated enhanced wound healing after a single dose of dexamethasone following post-dental extraction, with no related complications [38]. Notably, a single pediatric dose of dexamethasone has been shown to reduce the length of hospital stay. It is safe for use in pediatric patients, with no increase in blood glucose levels or delay in wound healing observed in strabismus surgery [39].

The aim of the present study was to develop a dual-drug-eluting suture capable of releasing both an antimicrobial and an anti-inflammatory agent, addressing the current gap in available sutures that can prevent surgical site infections and improve wound healing. We coated the suture with CHX and DEX using lauric acid as a carrier, without compromising its mechanical properties or cell culture biocompatibility. The dual-functionalized suture released both drugs and inhibited the growth of broad-spectrum bacteria, including drug-resistant strains such as MRSA, in addition to a common opportunistic yeast, *Candida albicans*, for several days.

## 2. Results

### 2.1. Sutures Were Dual-Coated with Chlorhexidine and Dexamethasone

To dual-coat the Vicryl sutures, they were dipped in a 2:1 chlorhexidine–dexamethasone solution using lauric acid as a carrier. To determine if the drugs were retained over the suture and verify the method’s consistency, a weight difference was calculated between uncoated and coated sutures (Table S1), resulting in a mean coating weight of 0.5 mg ± 0.2 mg (*n* = 6).

### 2.2. FTIR-ATR Characterization

**Figure 1:**
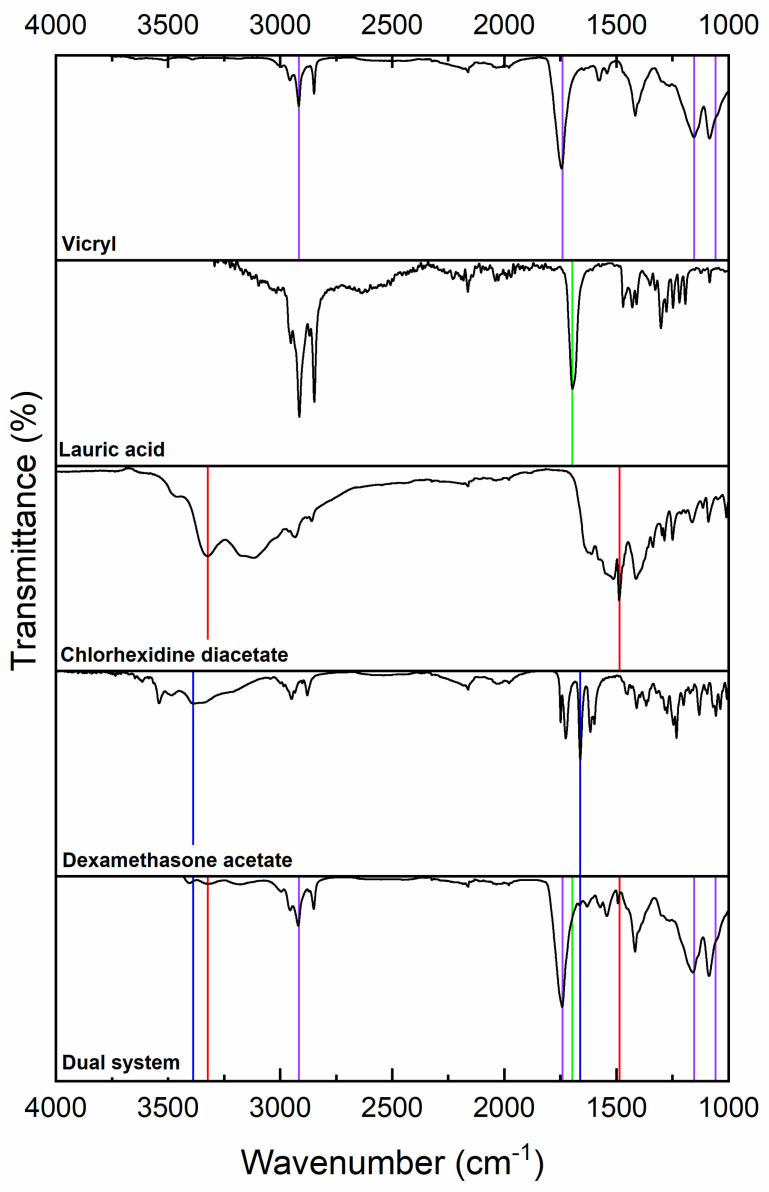
FTIR-ATR of dual-coated sutures, characteristic signals of the individual components are indicated in different colors: Vicryl suture (purple), lauric acid (green), chlorhexidine (red), and dexamethasone (blue).

A comparative infrared spectrum analysis was performed to confirm the dual CHX and DEX coating on the suture. The infrared spectrum analysis of untreated Vicryl showed a characteristic signal at 1740 cm^−1^ corresponding to the vibration of the ester-type carbonyl (C=O), in addition to a weak-intensity band at 2917 cm^−1^, corresponding to the C-H vibration (sp3 hybridization) of the Vicryl suture (purple lines in Figure 1) [40]. For lauric acid, an intense band at 1697 cm^−1^ corresponding to the C=O stretching of carboxylic acid was observed, in addition to a broad O-H stretching signal between 2800 and 2900 cm^−1^, also from carboxylic acid (green lines in Figure 1) [41]. In the case of chlorhexidine, a signal at 3323 cm^−1^ from the secondary amine group was observed, in addition to an intense signal at 1487 cm^−1^ due to N-H bending connected to the aromatic ring (red lines in Figure 1) [42]. For dexamethasone, a band was observed at 3389 cm^−1^ corresponding to O-H stretching, and at 1661 cm^−1^, a band corresponding to C=O stretching of the double bonds present in the cycle of dexamethasone was observed (blue lines in Figure 1) [43]. Dual-system spectra present the characteristic signals of each component, where the most predominant signals are those of the C=O stretches of the Vicryl suture (purple), in addition to the C-O stretches. We observed a signal from chlorhexidine at 3323 cm^−1^ N-H belonging to the secondary amine group, in addition to an intense signal from the N-H bending at 1487 cm^−1^ linked to the aromatic ring; this signal exhibits greater intensity in the suture of the dual-system, confirming the presence of chlorhexidine. For dexamethasone, we can observe O-H stretching present in the molecule in addition to stretches of sp3 hybridizations at 2879 cm^−1^ in the alkanes and at 2950 cm^−1^ of sp^2^ hybridization of the double bonds in the cycle. From the above results, the presence of CHX and DEX in the suture confirmed dual system integration.

### 2.3. Tensile Strength Results

**Figure 2:**
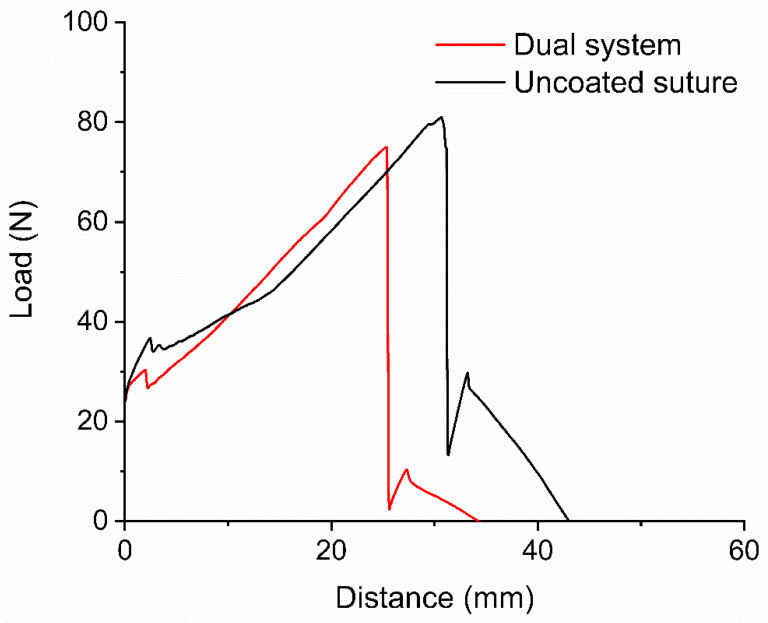
Comparative tensile strength between the uncoated suture and the dual-system suture.

To verify if the suture maintained its mechanical properties, which are essential for providing efficient wound closure support, a tensile strength test was conducted in a texture analyzer at break. Sutures exhibited a mean tensile strength of 79.81 and 76.93 N (Figure 2) for uncoated and coated sutures, respectively (Table 1 and Table S2), with no statistically significant difference (*p* = 0.487), indicating that the sutures’ mechanical properties remained unaffected.

### 2.4. Dual-System Quantification of Chlorhexidine and Dexamethasone

Qualitative analysis demonstrated that the chlorhexidine standard exhibited a retention time of 12.3 min, while dexamethasone showed a retention time of 18.4 min. In contrast, quantification of the drugs released from the sutures at 96 h revealed retention times of 12.8 min for chlorhexidine and 18.2 min for dexamethasone. The concordance in retention times between the reference standards and the analytes detected in the suture samples supports the chromatographic identification of both compounds and confirms their successful incorporation into the medical suture. Furthermore, the absorption spectra obtained from diode array detection for chlorhexidine and dexamethasone were consistent with values reported in the literature, thereby further supporting the qualitative identification of both compounds.

For quantitative determination, drug content in the sutures was calculated based on the area under the curve (AUC) of the chromatographic peaks using corresponding calibration curves (Figure S1). The cumulative amount released at 96 h was 28 μg (±1.17) for CHX and 13.29 μg (±0.657) for DEX. These values correspond to the total amount quantified in 1 mL of release medium from a 15 cm suture sample. The total drug takes into account the 5 mL of release medium (140 μg for CHX and 66.45 for DEX). 

### 2.5. Encapsulation Efficiency and Loading Capacity

Encapsulation efficiency and loading capacity were calculated using the drug release measures shown in Supplementary Materials. The resulting encapsulation efficiencies were 21.7% for CHX and 20.6% for DEX, with loading capacities of 5.6% and 2.7%, respectively, confirming the incorporation of the drugs within the lauric acid coating.

### 2.6. The Dual System Inhibits the Growth of Resistant Bacteria, MRSA

**Figure 3:**
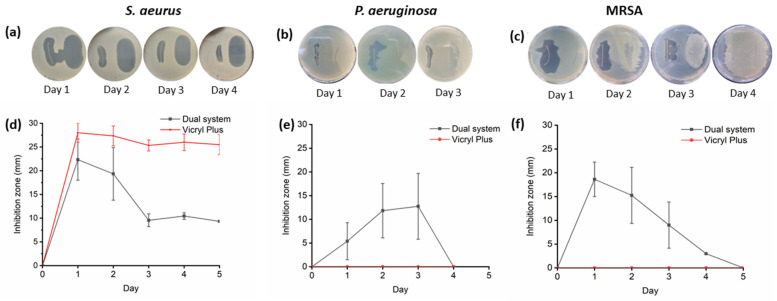
Inhibition zones per day in the presence of the dual system (left) and Vicryl Plus (right) on the agar plate against (**a**) *S. aureus*, (**b**) *P. aeruginosa*, and (**c**) MRSA. Graphic inhibition zones, per day in millimeters, of (**d**) *S. aureus*, (**e**) *P. aeruginosa*, and (**f**) MRSA.

**Figure 4:**
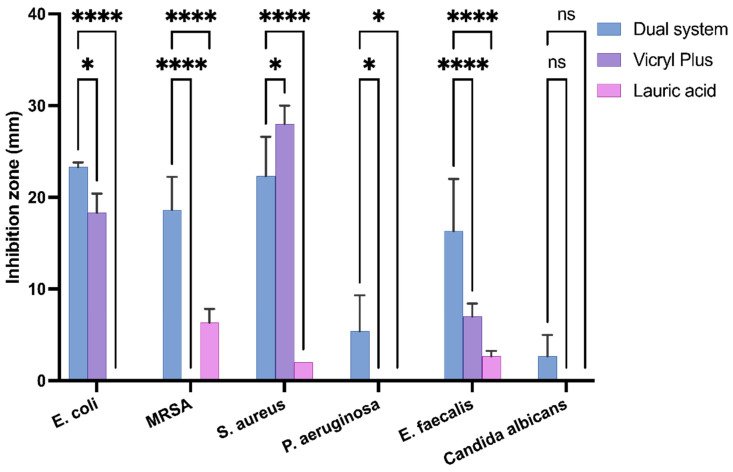
Inhibition Zones (mm) of Suture Formulations on Day 1 (mean ± SD). (*) indicated significant differences among groups (**** *p* < 0.0001; ns, not significant).

To evaluate the antimicrobial activity of the dual system, a bacterial growth inhibition test was conducted against the most common pathogens associated with surgical site infections. The results were compared with those obtained for Vicryl Plus and sutures containing lauric acid alone to evaluate their relative antimicrobial activity. Sutures of 3 cm were placed on a fresh seed agar plate containing Gram-positive bacteria (*Staphylococcus aureus* and *Enterococcus faecalis*), Gram-negative bacteria (*Escherichia coli* and *Pseudomonas aeruginosa*), and the fungus *Candida albicans*. The plate was incubated overnight at 37 °C. Inhibition zones were measured using a caliper and the same suture was transferred to a new, fresh seed agar plate daily until no inhibition zones were observed, as shown in Figure 3, Figures S2 and S3. Dual-system inhibition zones were observed in all strains; the largest inhibition zones were observed for *E. coli* and *S. aureus*. The dual system inhibited the growth of the tested microorganisms for at least 3 days as shown in Table S4, excluding *C. albicans* and *E. faecalis*. Statistical analysis confirmed significant differences between treatments (*p* < 0.05) for most bacterial strains on day 1 (Figure 4). In comparison, Vicryl Plus exhibits greater inhibition of *S. aureus* (28 mm) than the dual system (22 mm); however, the dual system showed superior activity against Gram-negative bacteria but was not effective against *C. albicans* and *P. aeruginosa*, the latter of which represents 16% of SSI cases [44].

To determine if lauric acid contributed independently to the antimicrobial effect, sutures coated only with lauric acid were evaluated against all the strains. Lauric acid exhibited a short antibacterial activity, with measurable inhibition observed only on Day 1 exclusively against Gram-positive bacteria, with no inhibition observed for Gram-negative strains or *Candida albicans*. This selective effect is consistent with the known susceptibility of Gram-positive bacteria to fatty acids [45]. In contrast, the dual system demonstrated significantly greater and more sustained inhibition across multiple bacterial species. Therefore, the broader antimicrobial spectrum observed in the dual system, particularly against *E. coli* and *P. aeruginosa*, cannot be explained by lauric acid alone and likely results from the complementary mechanisms of CHX that overcome these defenses.

To assess the dual system’s antimicrobial activity against antibiotic-resistant bacteria, an inhibition test was conducted against methicillin-resistant *Staphylococcus aureus* (MRSA). The dual system exhibited inhibited growth zones, initially 18 mm and subsequently 3 mm on the fourth day. In contrast, Vicryl Plus was ineffective, including on the first day. Significant inhibition was observed on day 1, with measurable antibacterial activity persisting through day 4, though at a reduced magnitude. These findings support the effectiveness of the dual system against antibiotic-resistant pathogens.

### 2.7. Cell Viability

**Figure 5:**
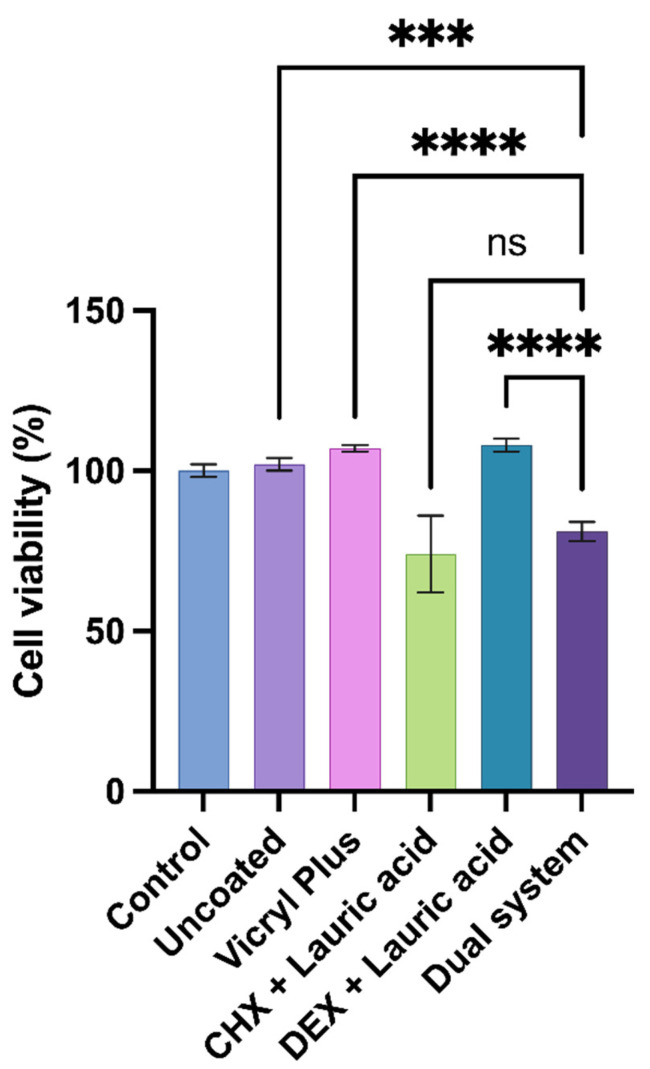
Cell viability of murine fibroblasts after 24 h exposure to coated and uncoated sutures measured by means of MTT assay (n = 4). The dual-system suture maintained cell viability above the 70%, indicating acceptable biocompatibility. (***) indicated significant differences among groups (*p* < 0.001); (****) *p* < 0.0001, and ns, not significant.

In vitro cytotoxicity was assessed to ensure cellular compatibility by measuring metabolic activity after exposing murine fibroblasts to eluates from the dual-system suture for 24 h. To determine the contribution of each component to overall toxicity, each agent was tested individually in the carrier and compared with the dual system. Controls, including eluates from untreated cells and an uncoated suture, were used as references. Following exposition, metabolic activity was measured using the MTT assay. The uncoated suture and the commercial Vicryl Plus exhibited metabolic activity above 100%. As the sutures were coated with the drugs, the metabolic activity was modified. The CHX plus lauric acid suture exhibited reduced viability (73.7%), suggesting chlorhexidine’s moderate cytotoxic effect. The DEX plus lauric acid suture sustained cell viability (108.4%) comparable to the Vicryl Plus suture, significant differences among groups (*p* < 0.001) are shown in Figure 5. The dual system exhibited acceptable metabolic activity (81.4%), with it being lower than the control but remaining above the 70%. It can therefore be considered safe for use in animal models.

## 3. Discussion

Our results confirmed that sutures can be coated with both drugs via a combined dip-coating process using lauric acid as a carrier. This process was previously reported by Obermier et al., who used fatty acids as effective carriers for sutures, coating them without altering their mechanical properties or decreasing the material’s surface roughness [46,47]. In this study, the weight variation between coated and uncoated sutures indicated consistent loading without compromising the physical integrity of the suture, as confirmed by the results of the tensile strength test. Compared to other drug-eluting suture production techniques, such as electrospinning or melt extrusion, dip coating does not require high temperatures or specialized equipment [18].

The incorporation and quantification of CHX and DEX were confirmed by characteristic spectral patterns in qualitative and quantitative analyses, in addition to FTIR and HPLC analyses, respectively. A dual HPLC quantification was conducted for CHX and DEX and confirmed the successful incorporation and elution of both drugs from the coated sutures. The retention times obtained for the reference standards were highly consistent with those observed in the release samples and demonstrate that the dual drug-eluting suture system maintains chemical integrity of both agents. Quantitative analysis demonstrated cumulative releases of 28 μg ± 1.17 mg for CHX and 13 μg ± 0.657 mg for DEX at 96 h comparable to the coating solution formulation consisting of 2:1 proportion, CHX and DEX respectively. The drug loading capacities indicate entrapment of both drugs, suggesting that the dual coating method is efficient, reproducible, and capable of releasing CHX and DEX. These findings support its potential as a multifunctional suture system that provides localized infection control and inflammation modulation while reducing the need for systemic drug administration [46].

The loaded CHX was sufficient to inhibit the growth of all microorganisms tested, including fungi and Gram-positive and negative bacteria. The largest inhibition zones were observed against *S. aureus* and *E. coli*, 22.3 mm and 23.3 mm respectively on the first day and 10.4 mm and 6.5 mm on the fourth day showing sustained antimicrobial effect over days. of particular note, the dual-coated system exhibited inhibition of growth even against a resistant microorganism, and the results of the commercial suture option, Vicryl Plus, were not in agreement with those of Frisch et al., who demonstrated efficient disinfection with a very low dose, namely, 0.05% CHX, before dental implantation or wound closure in humans [48].

Lauric acid alone has been reported to exhibit activity against viruses, bacteria, and fungi due to its surfactant properties, which destabilize cell walls. However the short inhibition and no activity against the Gram-negative may be caused by the properties of the membrane composed of lipopolysaccharides layers that limit the penetration of hydrophobic molecules such as fatty acids [49]. It has been suggested that *E. coli* can rapidly metabolize long-chain fatty acids, removing them before they can achieve inhibitory effect [50]. On the other hand, CHX is a lipophilic and positively charged molecule; these two properties enable the interaction of CHX with negatively charged phospholipids and lipopolysaccharides of the bacterial cell wall or the outer membrane [51]. These findings suggest that the antimicrobial performance of the dual system cannot be attributed exclusively to CHX and may result from the combined action of its incorporated active components for Gram-negative bacteria [52].

Conversely, dexamethasone has been reported to enhance fibroblast proliferation at pharmacologic concentrations (10^−6^–10^−5^ M) in vitro, and intraperitoneal administration in mice has demonstrated improvement in the wound healing process with low doses as low as 1 mg/kg (22–25 µg/day) [53,54]. Although the released DEX in the dual system appears to be at a low concentration, local administration via the suture provides direct exposure at the wound site. This local release could help maintain the balance between the inflammatory and reparative phases of wound healing, preventing excessive cytokine release while facilitating the necessary inflammatory activity that initiates tissue regeneration [29]. However, an evaluation of anti-inflammatory activity is required to establish subcytotoxic concentrations and ensure cell survival and biocompatibility in vivo.

The dual-system cytotoxicity test results demonstrated cell viability above 70%, confirming a non-cytotoxic effect in accordance with ISO 10993-5 [55]; this result is consistent with previous reports [56]. Cytotoxicity was evaluated at 24 h; however, the biological response to CHX is documented to be time- and dose-dependent and has been reported that induced cytotoxicity in fibroblasts increases as exposure is extended from 3 to 24 h [57]. In the specific case of coated sutures have observed that the antimicrobial and biological activity is most critical during the first 24 h [58]. Therefore, the results obtained at 24 h reflect a representative acute exposure scenario for the surrounding cells under standardized conditions.

These findings support the progression toward animal pre-clinical tests for anti-inflammatory and antimicrobial effects.

## 4. Materials and Methods

Vicryl (Ethicon Inc., Somerville, NJ, USA), a braided suture of Polyglactin 910, was employed in conjunction with chlorhexidine diacetate and dexamethasone acetate as antimicrobial and anti-inflammatory agents, respectively (Aldrich Chemical, Saint Louis, MO, USA). Lauric acid was used as a carrier for the drugs as previously reported [47]. Vicryl Plus, which is coated with triclosan (Ethicon Inc., Somerville, NJ, USA), was used as a reference for the inhibition zone test [46].

### 4.1. Suture Coating Solution

The suture coating process was conducted by means of dip coating, as reported by Obermier et al. [46], with modifications. Briefly, the coating solution was prepared by dissolving 100 mg of chlorhexidine diacetate in 10 mL of 96% ethanol in a screw-cap container, followed by homogenization with magnetic stirring. Thereafter, 237 mg of lauric acid was added, followed by 50 mg of dexamethasone acetate; the mixture was stirred until complete dissolution.

### 4.2. Suture Coating Process

The suture was divided into 15 cm segments and individually weighed (*n* = 3) using an analytical balance. Subsequently, each thread was submerged in the coating solution for 10 min, using an Orbital Shaker (VWR Shel Lab, Cornelius, OR, USA), set to 37 °C and shaking at 150 rpm. Following the incubation period, the samples were air-dried overnight at room temperature within a Petri dish and subsequently reweighed using an analytical balance to determine the weight difference, which was used to calculate the encapsulation and loading efficiency.

### 4.3. FTIR-ATR Spectroscopy Analysis of Coated Sutures

Infrared characterization of the sutures was performed using a Fourier-transform infrared (FTIR) spectrophotometer (Nicolet iS5, Thermo Scientific, Madison, WI, USA), to confirm the load. Uncoated and coated sutures were identified using an ATR module in the range of 4000 to 900 cm^−1^, with 16 scans applied.

### 4.4. Tensile Strength Test

Sutures of 15 cm in length were tested using grip fixtures (TA-DGF) to apply tension load with the texture analyzer (Brookfield CT3, Middleboro, MA, USA), in a standard atmosphere (65% RH, ki25 °C).

### 4.5. Coated Suture Drug Release

Sutures were incubated in 5 mL of methanol at 250 rpm on an oscillator at 37 °C for 96 h in triplicate.

### 4.6. HPLC Quantification of Chlorhexidine Diacetate and Dexamethasone Acetate

The study was performed using an UltiMate™ 3000 HPLC Chromatograph (Thermo Fisher Scientific, Germering, Germany), equipped with a C18 column (250 mm × 4.6 mm, 5 µm particle size). Both drugs were analyzed simultaneously using a validated qualitative and quantitative method. The chromatographic conditions were established based on previously reported literature [59,60]. Briefly, a gradient elution method was employed at a flow rate of 1.0 mL/min and a column temperature of 30 °C. Detection was carried out at 240 nm for dexamethasone acetate and 260 nm for chlorhexidine diacetate. Four mobile phases were used: methanol (MeOH), water, acetonitrile, and a triethylamine buffer solution (7.5 mL of triethylamine diluted to 1000 mL with water and adjusted to pH 3.0 using phosphoric acid). All mobile phases were filtered through a 0.22 µm nylon membrane and degassed prior to use. The detailed gradient program is described on Table S3.

Samples from the 96 h release medium were collected and filtered through a 13 mm Acrodisc^®^ nylon syringe filter (Pall Corporation, Port Washington, NY, USA), with a 0.2 µm pore size. A 1 mL aliquot of the filtered sample was transferred into a 2 mL Thermo Scientific™ TTR-T vial (Rockwood, TN, USA), for injection into the HPLC system. Qualitative analysis was performed based on retention times and absorption spectrum scanning using a diode array detector (DAD). Quantitative analysis was conducted by calculating the area under the curve (AUC) of the corresponding analytical peaks in the obtained chromatograms.

### 4.7. Dual Drug-Eluting Suture Antimicrobial Effect

To assess the antimicrobial efficacy of the sutures coated with the dual system and sutures with lauric acid alone, they were evaluated against *Staphylococcus aureus* (ATCC 29213™), a methicillin-resistant *S. aureus* (MRSA) clinical isolate from a public healthcare facility in Tijuana, *Enterococcus faecalis* (NCTC 775™), *Escherichia coli* (ATCC 25922™), *Pseudomonas aeruginosa* (ATCC 27853™), and *Candida albicans* (ATCC 10231). Suture tests were conducted in agar plates to compare to Vicryl Plus [47]. Briefly, within a Class II biohazard cabinet, 3 cm suture samples were placed on Mueller–Hinton II Agar plates, which were inoculated with a bacterial suspension at a 0.5 McFarland standard. The plates containing the samples were then incubated overnight at 37 °C. Subsequently, the zones of inhibition were measured using a caliper, and the coated suture samples were transferred to freshly inoculated agar plates. This process was repeated until the sutures no longer exhibited inhibitory effects. All of the experiments were performed in triplicate to ensure reproducibility.

### 4.8. Indirect Cell Viability Assay

Murine fibroblast cells BALB/3T3 and murine embryonic fibroblasts (ATCC CCL-163, Manassas, VA, USA) were used and seeded at a density of 10,000 cells per well in 96-well plates containing DMEM and incubated for 24 h at 37 °C and 5% CO_2_. Before testing, sutures were sterilized under UV light and immersed in 1.5 mL of DMEM in 2 mL tubes. The tubes were then incubated at 37 °C and 150 rpm for 24 h to obtain the elution medium. After incubation, 100 µL of each elution medium was transferred to 96-well plates containing fibroblasts and incubated for 24 h. Cell metabolic activity was then evaluated using the Cell Proliferation Kit I (MTT; Roche Diagnostics GmbH, Mannheim, Germany) according to the manufacturer’s instructions. Absorbance was measured using a Multiskan FC microplate reader (Thermo Fisher Scientific, Waltham, MA, USA). Cell viability was expressed as a percentage relative to the control group. All experiments were carried out in quadruplicate.

## 5. Conclusions

In this study, we successfully developed and characterized a dual-drug-eluting suture system that releases chlorhexidine and dexamethasone using lauric acid as an anchor. The coating process preserved the mechanical integrity of the suture while enabling sustained drug release over several days. Antimicrobial assays demonstrated broad-spectrum efficacy against diverse microorganisms, including Gram-positive and -negative bacteria and fungi. Of clinical relevance, the dual-system suture inhibited the growth of a resistant strain of MRSA and *Candida albicans*. Furthermore, cytotoxicity tests confirmed acceptable biocompatibility, in compliance with ISO 10993-5 standards [55].

The integration of both antimicrobial and anti-inflammatory agents into a single suture represents a promising strategy for preventing surgical site infections and modulating local inflammation. These findings support the potential of dual-functionalized sutures as a multifunctional tool in surgical practice. Further *in vivo* studies are warranted to evaluate long-term biocompatibility, anti-inflammatory performance, and clinical applicability.

## Figures and Tables

**Table 1:** The sutures’ pre- and post-coating tensile strength.

	Uncoated Suture	Dual-System
Mean	79.81 N	76.93 N
Standard Desv. ^1^	5.08	4.09

^1^ Mean values for uncoated and coated sutures (*n* = 3).

## Data Availability

The original contributions presented in this study are included in the article/Supplementary Materials.

## Supplementary Materials

The following supporting information can be downloaded at: https://www.mdpi.com/article/10.3390/molecules31071200/s1: Table S1. Suture’s weight before/after coating; Table S2. The sutures’ pre- and post-coating tensile strength; Table S3. The detailed gradient program for dual quantification of CHX and DEX; Table S4. Antimicrobial activity of the sutures; Figure S1. Chromatograms of the (a) standard solution of CHX and DEX and (b) elution of suture after 96 h; Figure S2. Inhibition of sutures against *E. faecalis* NCTC 775; Figure S3. Inhibition of sutures against *E. coli* ATCC 25922. Inhibition days from 1 to 4; Figure S4. Days of inhibition against *Candida albicans* ATCC 10231.

## Abbreviations

The following abbreviations are used in this manuscript:

SSI	Surgical Site Infections
CHX	Chlorhexidine
DEX	Dexamethasone
