# Supplementary material for: Dual-Action Sutures: Chlorhexidine and Dexamethasone for Infection Control and Inflammation Suppression
Source: Molecules. 2026 Apr 4;31(7):1200. doi: 10.3390/molecules31071200 (PMC13074332; doi:10.3390/molecules31071200)
Supplement: Supplementary file 1 [file molecules-31-01200-s001.zip › molecules-4142372-supplementary.pdf]

Dual-Action Sutures: Chlorhexidine and Dexamethasone for Infection Control and Inflammation Suppression

Supplementary S1

Table S1. Suture's weight before/after coating.

Suture	Initial weight (mg)	Final weight (mg)	Weight Difference
1	26.3	26.9	0.6
2	26.9	27.5	0.6
3	26.3	26.4	0.1
4	26.3	26.9	0.6
5	26.1	26.7	0.6
6	27.3	27.8	0.5
**Mean**	26.5	27.0	0.5
**Std Dev**	0.46	0.52	0.20

Supplementary S2

Drug Fraction, Theoretical Drug Loading, Encapsulation Efficiency, and Loading Capacity

The drug fraction in the coating mixture was determined as the ratio of the mass of each active compound to the total mass of solids in the coating formulation. The coating solution consisted of chlorhexidine diacetate (CHX), dexamethasone acetate (DEX), and lauric acid, with a total solid content of 387 mg. The mass fractions of each drug were calculated using equation 1:

$$\text{Drug fraction (\%)} = (\text{Mass of drug (mg)}) / (\text{Total mass of solids(mg)}) \times 100, \quad \text{(S1)}$$

According to this formulation, the resulting drug fractions were 25.84% for CHX and 12.92% for DEX, which were subsequently used to estimate the theoretical drug loading per suture.

The mean increase in coating weight per suture (15 cm) was 0.5 mg, determined by weighing uncoated and coated sutures. Theoretical drug loading (TDL) was calculated according to equation 2:

$$\text{TDL (mg)} = \text{Coating weight (mg)} \times \text{Drug fraction in coating}, \quad \text{(S2)}$$

The calculated theoretical load corresponded to 0.1292 mg (129.2 μg) of CHX and 0.0646 mg (64.6 μg) of DEX per 15 cm suture, equivalent to 8.61 μg/cm and 4.31 μg/cm, respectively, used to calculate the encapsulation efficiency (EE%) and loading capacity (LC%). The EE% represents the ratio between the actual amount of drug incorporated in the coating (determined by HPLC) and the TDL expected, as shown in equation 3:

$$\text{EE (\%)} = (\text{Actual amount of drug in sample(mg)}) / (\text{Theoretical amount of drug in the sample (mg)}) \times 100 \quad \text{(S3)}$$

The LC% expresses the proportion of drug relative to the total mass of the coated sample, calculated using equation 4:

$$\text{LC (\%)} = (\text{actual amount of drug in sample (mg)}) / (\text{amount of sample(mg)}) \times 100 \quad \text{(S4)}$$

These parameters were calculated using the drug quantities released after 96 h as the experimentally measured values. The resulting encapsulation efficiencies were 21.7% for CHX and 20.6% for DEX, with loading capacities of 5.6% and 2.7%, respectively, confirming effective drug incorporation within the lauric acid coating.

Supplementary S3

Table S2. The sutures' pre- and post-coating tensile strength

Uncoated (n)	Peak Load (N)
1	74.24
2	81
3	84.18
Average	79.81
Dual system (n)	Peak Load (N)
1	74.96
2	81.63
3	74.2
Average	76.93

Supplementary S4

Table S3. The detailed gradient program for dual quantification of CHX and DEX

Time (min)	MeOH (%)	Water (%)	Acetonitrile (%)	Buffer (%)
0	0	0	28	72
1	0	0	28	72
12	0	0	28	72
12.5	10	0	40	50
13.5	10	0	50	40

14.5	20	0	60	20
15.5	30	10	50	10
16.5	40	10	50	0
17.5	50	20	30	0
18.5	70	30	10	0
19.5	65	35	0	0
20	0	0	28	72
23	0	0	28	72

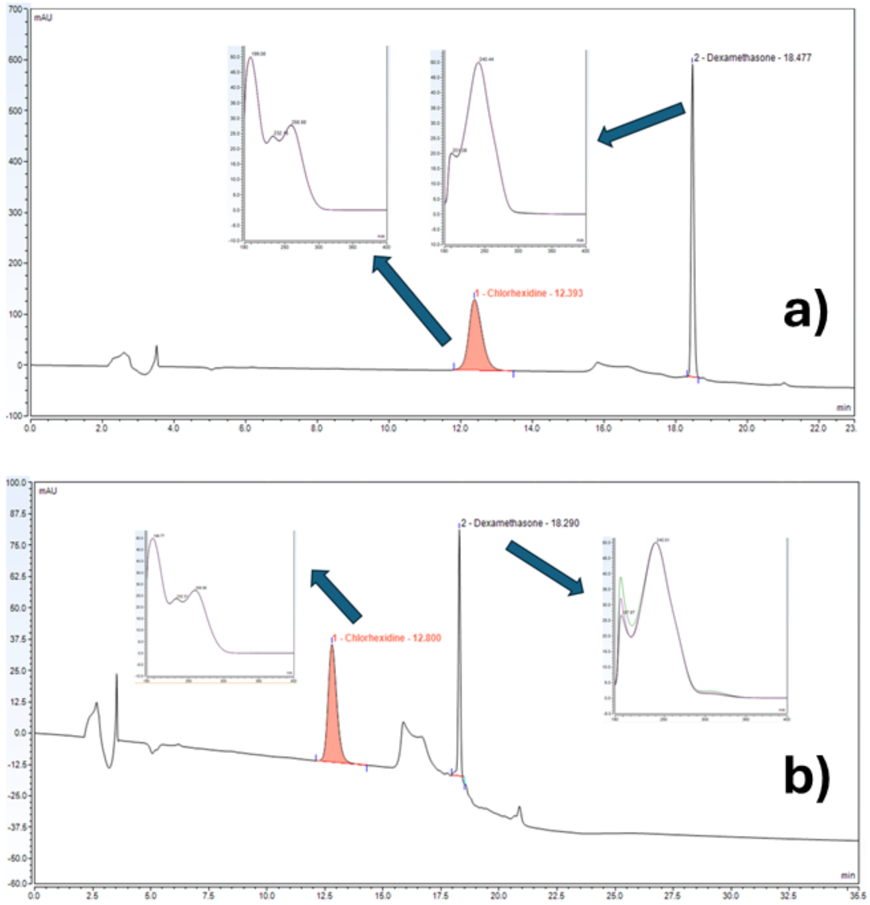

Figure S1. Chromatograms of the (**a**) standard solution of CHX and DEX and (**b**) elution of suture after 96 h.

Supplementary S5

Table S4. Antimicrobial activity of the sutures.

		Dual system	Vicryl Plus	Lauric acid
Microorganism	Day	Inhibition zone (mm)	Inhibition zone (mm)	Inhibition zone (mm)
S. aureus	1	22.3 ± 4.3	28.0 ± 2.0	2.00 ± 0.00
	2	19.3 ± 5.5	27.3 ± 2.1	0.00 ± 0.00
	3	9.6 ± 1.3	25.3 ± 1.2	—
	4	10.4 ± 0.7	26.00 ± 1.7	—
	5	9.3 ± 0.0	25.50 ± 2.1	—
E. faecalis	1	16.3 ± 5.7	7.00 ± 1.4	2.7 ± 0.6
	2	16.7 ± 6.4	0.00 ± 0.00	0.0 ± 0.0
E. coli	1	23.3 ± 0.5	18.3 ± 2.1	0.00 ± 0.00
	2	14.7 ± 1.7	17.00 ± 0.0	—
	3	8.8 ± 5.9	15.7 ± 0.6	—
	4	6.5 ± 3.9	15.00 ± 1.0	—
P. aeruginosa	1	5.4 ± 3.9	0.00 ± 0.0	0.00 ± 0.00
	2	11.8 ± 5.7	—	—
	3	12.8 ± 6.9	—	—
Candida albicans	1	2.7 ± 2.3	0.00 ± 0.0	0.00 ± 0.00
Methicillin-resistant *S. aureus*	1	18.6 ± 3.6	0.00 ± 0.0	6.33 ± 1.5
	2	15.3 ± 5.9	—	0.00 ± 0.0
	3	9.0 ± 4.9	—	—
	4	3.00 ± 0.00	—	—

Data expressed as mean ± SD (mm). (—) indicates no available measurement.

Supplementary S6

1.1. Sensitivity of Enterococcus fecalis NCTC 775 to the Dual System

The inhibition zones against *E. faecalis* were larger with the dual system compared to the triclosan-coated suture (Figure S2). However, in both cases, the inhibition appears visually small in both systems.

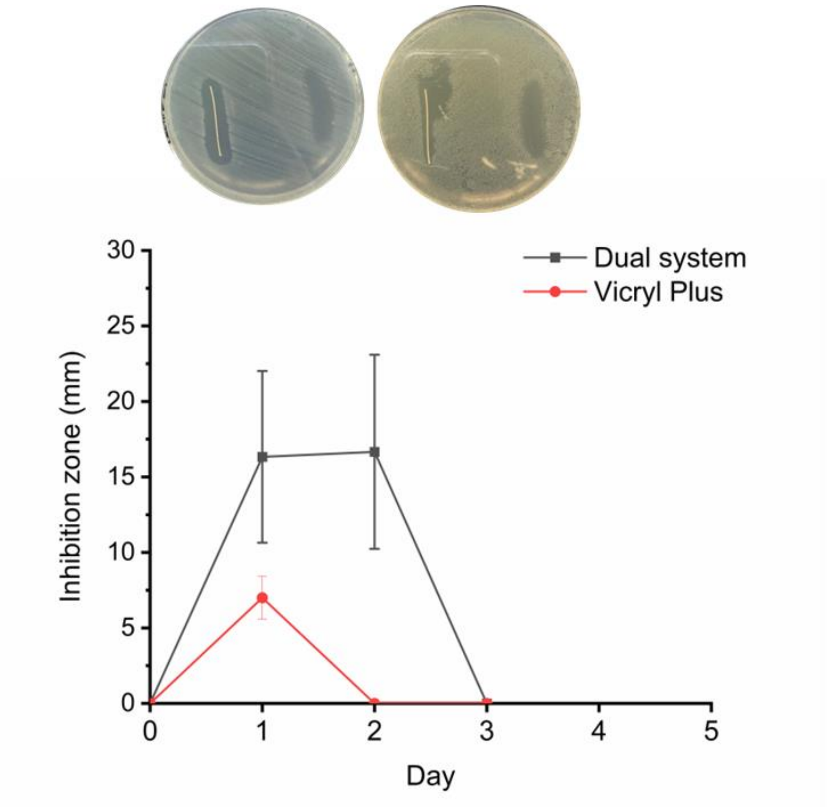

Figure S2. Inhibition of sutures against *E. faecalis* NCTC 775. Dual system on the left and Vicryl Plus on the right.

1.2 Sensitivity of Escherichia coli ATCC 25922 to the Dual System

The inhibition of the suture with a dual system was compared using the commercial triclosan-coated suture as a reference against *E. coli* ATCC 25922. It was observed that the inhibition zone of triclosan was slightly smaller on the first day but remained similar over time. In contrast, the suture with the dual system initially exhibited a large inhibition zone but did not maintain it over time. As shown in Table 4, from day 3 onward, the inhibition zone of the dual system decreased drastically to approximately half of that observed on day 2 (Figure S3).

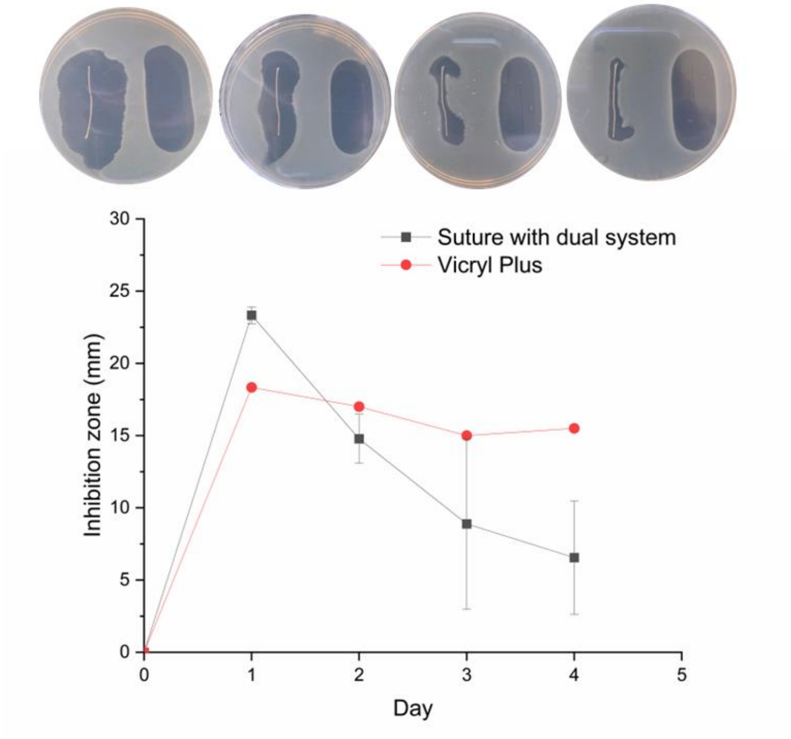

Figure S3. Inhibition of sutures against *E. coli* ATCC 25922. Inhibition days from 1 to 4. Left: Dual-system suture. Right: Vicryl Plus.

1.3 Sensitivity of Candida albicans ATCC 10231 to the Dual System

A test was conducted to verify the ability of the dual system to inhibit *C. albicans* ATCC 10231, using the commercial triclosan-coated suture as a reference. The results did not show a clearly visible inhibition (Figure S4). However, inhibition zones were present in the dual-system sutures, whereas no inhibition was observed in the triclosan-coated suture, making this coating promising.

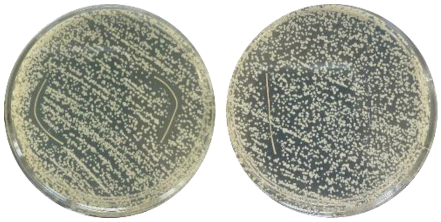

(**a**) (**b**)

Figure S4. Days of inhibition against *Candida albicans* ATCC 10231. Inhibition of sutures with a dual system in triplicate (**a, b**) and triclosan-coated suture (**b**) on the right side.
